# Get More Out of Your Data: A New Approach to Agglomeration and Aggregation Studies Using Nanoparticle Impact Experiments

**DOI:** 10.1002/open.201300005

**Published:** 2013-03-15

**Authors:** Joanna Ellison, Kristina Tschulik, Emma J E Stuart, Kerstin Jurkschat, Dario Omanović, Margitta Uhlemann, Alison Crossley, Richard G Compton

**Affiliations:** [a] Department of Chemistry, Physical & Theoretical Chemistry Laboratory, Oxford UniversitySouth Parks Road, Oxford OX1 3QZ (United Kingdom) E-mail: kristina.tschulik@chem.ox.ac.ukrichard.compton@chem.ox.ac.uk; [b] Department of Materials, Oxford University, Begbroke Science ParkSandy Lane, Yarnton OX5 1PF (United Kingdom); [c] Center for Marine and Environmental Research, Ruđer Bošković InstitutePOB 180, 10001 Zagreb (Croatia); [d] IFW Dresden, Institute for Complex MaterialsP.O. Box 270016, 01171 Dresden (Germany)

**Keywords:** agglomeration and aggregation states, anodic particle coloumetry, electrochemistry, nanoparticle impact experiments, nanoparticle tracking analysis

## Abstract

Anodic particle coloumetry is used to size silver nanoparticles impacting a carbon microelectrode in a potassium chloride/citrate solution. Besides their size, their agglomeration state in solution is also investigated solely by electrochemical means and subsequent data analysis. Validation of this new approach to nanoparticle agglomeration studies is performed by comparison with the results of a commercially available nanoparticle tracking analysis system, which shows excellent agreement. Moreover, it is demonstrated that the electrochemical technique has the advantage of directly yielding the number of atoms per impacting nanoparticle irrespective of its shape. This is not true for the optical nanoparticle tracking system, which requires a correction for the nonspherical shape of agglomerated nanoparticles to derive reasonable information on the agglomeration state.

## Introduction

Nanoparticles are not only of major scientific interest, due to the potential of tuning their properties by changing their size, chemical composition or capping agent. They are also used in a vast variety of consumer products, utilizing these specifically adjusted properties and the fact that, compared to bulk products, material costs can be significantly reduced in many cases. The application of silver nanoparticles (Ag NPs) is one of the most prominent examples of this, employing the antibacterial effect of silver while keeping the total amount of this noble metal at a minimum. Today, several hundred commercial products containing Ag NPs can be found on the market, for example in health (medical instruments, tooth paste), fitness (functional clothing) and food industries (water sanitizer).^1^ This omnipresence and the resulting significant release into the environment, requires reliable and affordable detection and characterisation of these nanoparticles. Here not only their size,^2^ but also their agglomeration and aggregation state is of major importance, since these greatly affect, for example, the impact of nanoparticles on environmental and biological systems. Obviously, agglomeration depends on the stabilizing agent of the Ag NPs and the composition of the surrounding media, making proper predictions for agglomeration and aggregation extremely difficult. Therefore, intensive field studies are required to improve knowledge of nanoparticle influences on biological and environmental systems.

**Figure 1:**
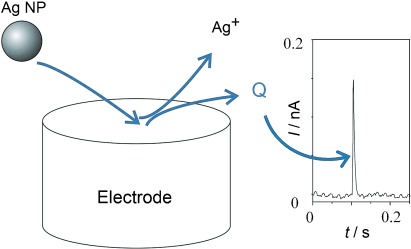
Schematic drawing of the APC working principle, showing the impact of a Ag NP and its oxidation to yield a size-specific current spike of charge Q.

The most common methods for detecting nanoparticles and analysing their size and agglomeration state in different solutions are dynamic light scattering (DLS) and nanoparticle tracking analysis (NTA). Additionally, electrochemical sizing of nanoparticles during their impacts at microelectrodes is possible, either by detecting reactions occurring at the nanoparticle surface^3^, ^4^ or by oxidising the NP itself.^5^ The latter is known as anodic particle coloumetry (APC) and uses the anodic current that results from oxidation of a nanoparticle during its impact at a microelectrode. The contact time of nanoparticles at the electrode is in the range of 1 ms to 20 ms.^6^, ^7^ Thus, it enables oxidation of the NP and detection of the resulting anodic current as a spike in the current transient. The working principle is schematically visualised in Figure 1.

Because this is a purely electrochemical method, it allows the user to distinguish between different chemical species in a mixture,^8^ and, in contrast to NTA and DLS, it can be used in opaque solutions as no optical signal needs to be detected. In complex matrices, for example, algae-containing water samples, this is very beneficial as it reduces the effort of sample pretreatment. Most studies so far have been focused on the use of APC to gain information on the particle size.^4^, ^5^ In contrast, agglomeration has been studied to a smaller extent.^9^ However, as stated above, to predict the effect of nanoparticles on a variety of biological and environmental systems, the different agglomeration and aggregation states in a specific solution need to be known in more detail than we do today. Thus, we suggest a new approach to derive this additional information by analysing APC data in light of both the analysis of the nanoparticle (monomer) size and the agglomeration state^1^ in a specific solution. To validate this approach and the derived data, comparative NTA analysis is performed.

## Results and Discussion

### Voltammetric Ag NP stripping

**Figure 2:**
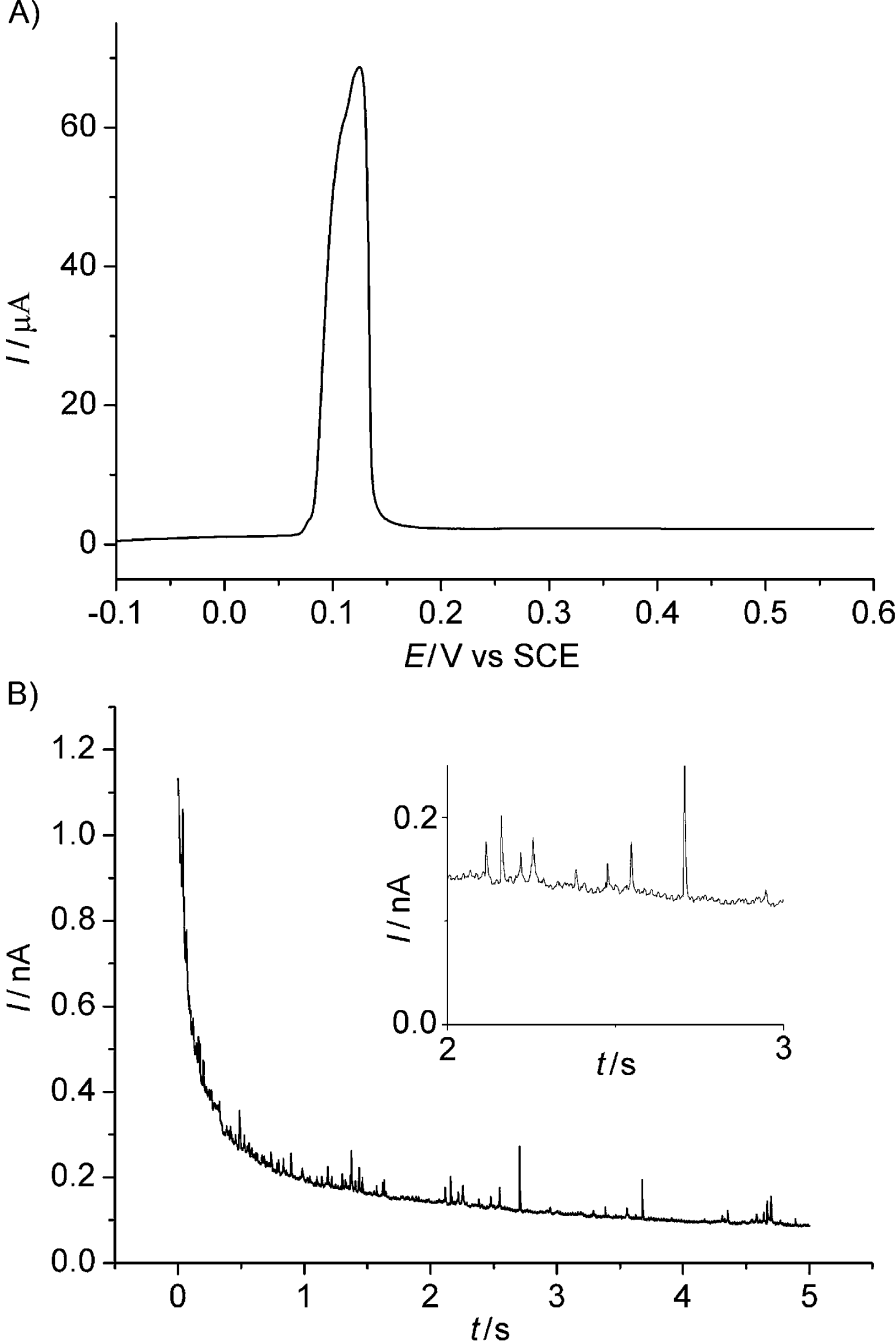
A) Stripping voltammogram of a Ag NP-modified GC macro electrode (sweep rate=0.02 V s^−1^) and B) chronoamperogram recorded during APC experiments (*E*=0.3 V), anodic current spikes show oxidation of Ag NPs when impacting the WE. Experiments were performed in a solution of KCl (0.09 m) and NaC_6_H_7_O_7_ (0.01 m).

The recorded linear sweep stripping voltammogram plotted in Figure 2 a shows that stripping of silver from a Ag NP-modified glassy carbon (GC) electrode in a potassium chloride/citrate electrolyte starts at an approximate value of 0.1 V versus saturated calomel electrode (SCE). Except for the sharp silver stripping peak, no additional peaks were found in the potential region up to 0.6 V, confirming the absence of side reactions in this potential region.

### Impact experiments

Based on the voltammetric stripping experiments, a potential of 0.3 V was chosen for the chronoamperometric impact experiments to assure oxidation of Ag NPs when contacting the working electrode (WE):^9^


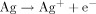
(1)

Figure 2 b exemplarily shows one of the 115 obtained chronoamperograms. Ag NPs impacting the WE are oxidised, causing anodic spikes in the current. The charge (*Q*) assigned to each spike is the integral of the current (*I*) over time (*t*)


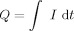
(2)

and the duration of a single spike is in the order of 1 ms to 20 ms.^6^, ^7^ This anodic charge is linked with the amount of oxidised silver by Faraday′s law:


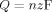
(3)

where *n* is moles of silver, *z* is the number of exchanged electrons per oxidised silver atom (*z*=1; see [Eq. (1)]) and F is the Faraday constant (96 485 C mol^−1^).

Because one electron is generated per oxidised silver atom, the charge (*Q*) assigned to each spike can directly be used to calculate the number of atoms (*N*) forming the impacting nanoparticle, taking the elementary charge *e*=1.602×10^−19^ C:


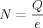
(4)

In the following, we assume that NP impact and APC experiments, like the majority of stochastic processes, obey the statistics of a normal distribution, that is, the detected charge, and hence the number of atoms, follows a Gaussian. The peak position is defined by the mean (expected value) for the number of atoms in a particle (*N*), and the full width at half maximum (FWHM) is determined by the standard deviation (*σ*).

**Figure 3:**
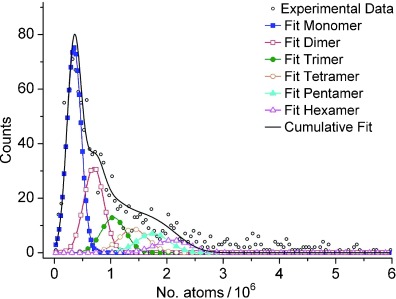
Experimentally obtained distribution of the NP size in the KCl/citrate electrolyte, as detected by APC (○). The data fit (—) is derived by overlying the individual Gaussians for NP monomers (▪), dimers (□), trimers (•), tetramers (○), pentamers (▴) and hexamers (▵).

To enable fitting of the experimental data, a histogram was drawn, containing the calculated number of atoms for all detected 1333 impacts (see Figure 3, ○). Finding a proper bin size is crucial for this. The smaller the bin size, the more precisely it describes the real distribution, but the corresponding decrease of counts per bin limits the stochastic description. The large number of analysed spikes allowed us to set the bin size to 0.05×10^6^ atoms, while still being able to fit the data appropriately. The bin centres and the accompanied number of impacts are displayed in Figure 3 (○). The fact that this distribution is not a Gaussian can be related to agglomeration of NP monomers, which yields a convolution of several individual Gaussians, as will be discussed in the following section.

Agglomeration and aggregation of particles describes the (reversible or permanent) sticking of at least two monomers. Consequently, the number of atoms of an agglomerate is an integer multiple of the number of atoms of a monomer (*N*_M_). For example, the number of atoms of a dimer (*N*_2M_) and a trimer (*N*_3M_) is twice and three times that of a monomer, respectively.


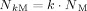
(5)

with k denoting the number of monomers in a nanoparticle agglomerate.

For populations following a normal distribution, not only the mean values but also their standard deviations, that is, their FWHM, are directly linked. Consequently, the Gaussians for NP agglomerates (*N*_*k*M_, *σ*_*k*M_) can be derived from those of the NP monomer (mean=*N*_M_, FWHM=standard deviation=*σ*_M_):


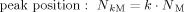
(6)


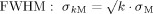
(7)

Applying these correlations to the results of the impact experiments, we can deconvolute the data into the individual Gaussians. Thus, both information about NP monomers and also about the size and relative amount of agglomerates can be accessed. In Figure 3 the experimental data was fitted on this basis, showing the presence of NP monomers (*N*_M_), dimers (*N*_2M_), trimers (*N*_3M_), tetramers (*N*_4M_), pentamers (*N*_5M_) and hexamers (*N*_6M_). It should be noted that except for the intensity of each agglomerate, which describes its relative concentration (see below), no additional parameters were included in the fitting, since means and FWHM values for each agglomerate are defined by [Eq. (6) and (7)]. The good quality of the resulting fit indicates the validity of this new approach of APC analysis and demonstrates that more information about the nanoparticle agglomeration state in a specific solution can be extracted from electrochemical impact data than previously realised.^9^

The obtained mean for the number of atoms of a monomer (*N*_M_) is 0.35⋅10^6^ atoms. Assuming spherically shaped NP monomers, this value is linked to the radius of a single nanoparticle (*r*_M_), according to:


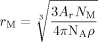
(8)

where *A*_r_ is the relative mass of silver atoms (*A*_r_(Ag)=107.87 g mol^−1^); N_A_ is the Avogadro constant (6.022×10^23^ mol^−1^) and *ρ* is the density of silver (*ρ*(Ag)=10.49 g cm^−3^).

Thus, the impact experiments yield a radius of *r*_M_=11.3±0.6 nm for a single Ag NP, which is in excellent agreement with the results obtained by SEM and NTA in water/citrate (see Experimental Section).

### NTA studies

To validate the APC results, NP agglomeration in the KCl/citrate electrolyte was also analysed by NTA using a NanoSight (LM 10, NanoSight Ltd.). This device tracks Brownian motion-driven particle movements in 2 D and assigns a diffusion coefficient (D) to each NP based on its individual displacement per time frame (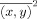).^11^


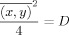
(9)

**Figure 4:**
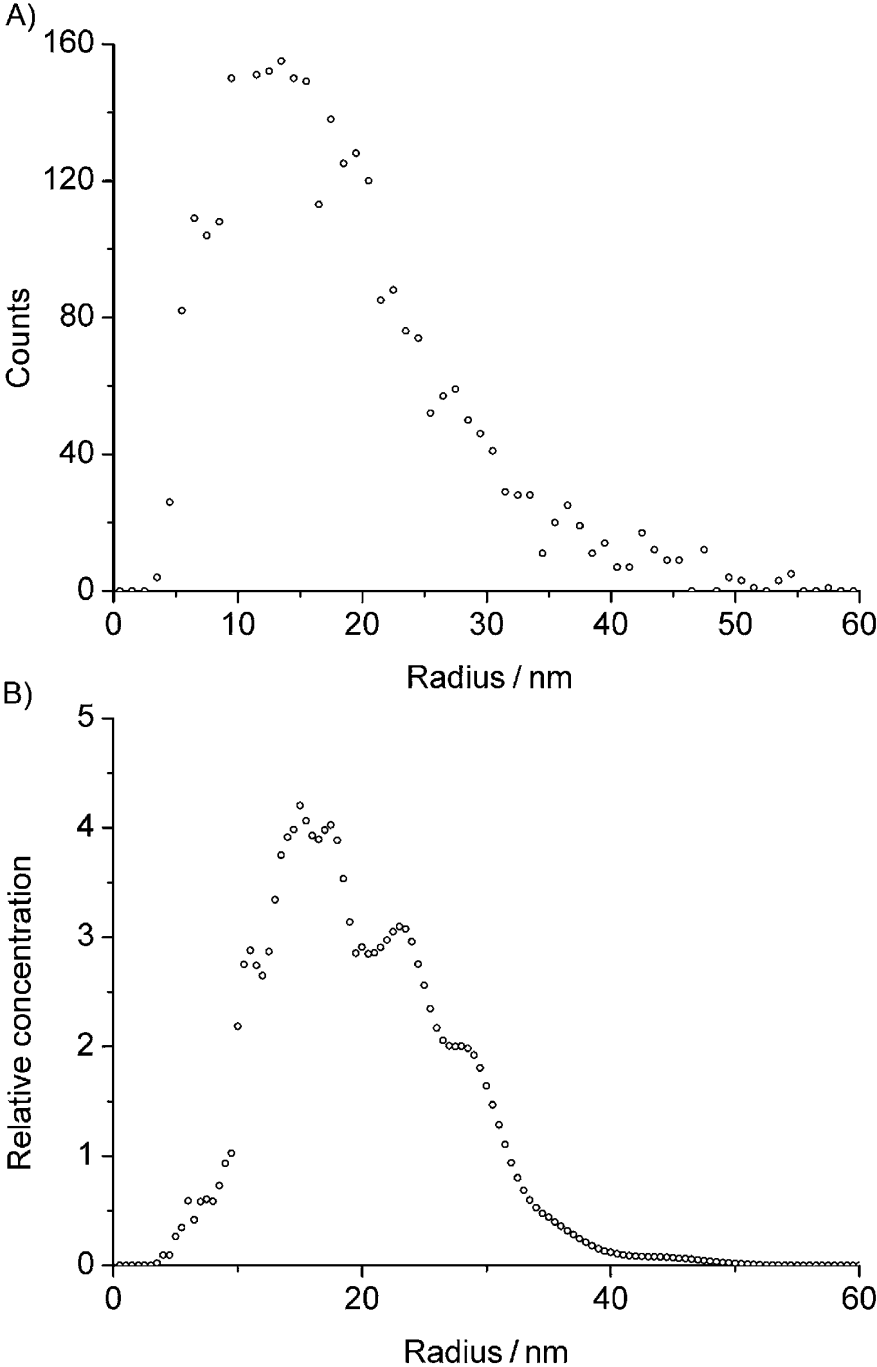
Size distribution of the Ag NP in KCl/citrate solution, as determined by nanoparticle tracking analysis (NanoSight). A) Number of tracked NPs over their size (calculated from their *D* values) and B) relative concentration of NPs over their size (output data, fitted automatically by the NTA software); assuming spherically shaped NPs.

Afterwards, the Stokes–Einstein relation is employed to calculate a particle radius, taking the electrolyte viscosity (*η*=1.002×10^−3^ kg m^−1^ s^−1^ at 293 K^12^) and temperature (*T*=293 K) into account and assuming a spherical particle shape.^11^


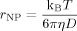
(10)

where k_B_ is the Boltzmann constant (1.38×10^23^ kg m^2^ s^−2^ K^−1^). This is done by the integrated NanoSight software for every NP that has been tracked for at least five times (this threshold value is automatically adjusted by the system depending on the size of the detected NPs and is meant to reduce measurement errors). Hence, a radius is assigned to each of these NPs, yielding the raw distribution shown in Figure 4 a. The internal NTA software fits this data assuming a spherical particle shape and introducing a sizedependent weighting factor. The resulting output data is plotted in Figure 4 b and, in the authors’ opinion, does not satisfactorily reproduce the raw data shown in Figure 4 a.

Consequently, we adapted the analysis of the NTA data according to our needs for agglomeration studies, as described below. Still assuming a spherical shape, the experimentally derived diffusion coefficient (*D*) can be used to determine the number of atoms (*N*) forming a NP:


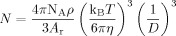
(11)

**Figure 5:**
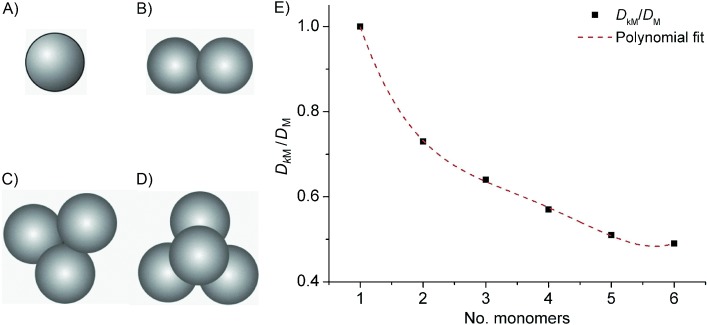
Schematic drawings of a spherical nanoparticle monomer (A) and nonspherical agglomerates formed by several monomers, for example, dimer (B), trimer (C) and tetramer (D). Polynomial extrapolation of the correction factors given in Ref. 13 was performed to obtain values for pentamers and hexamers (E).

**Figure 7:**
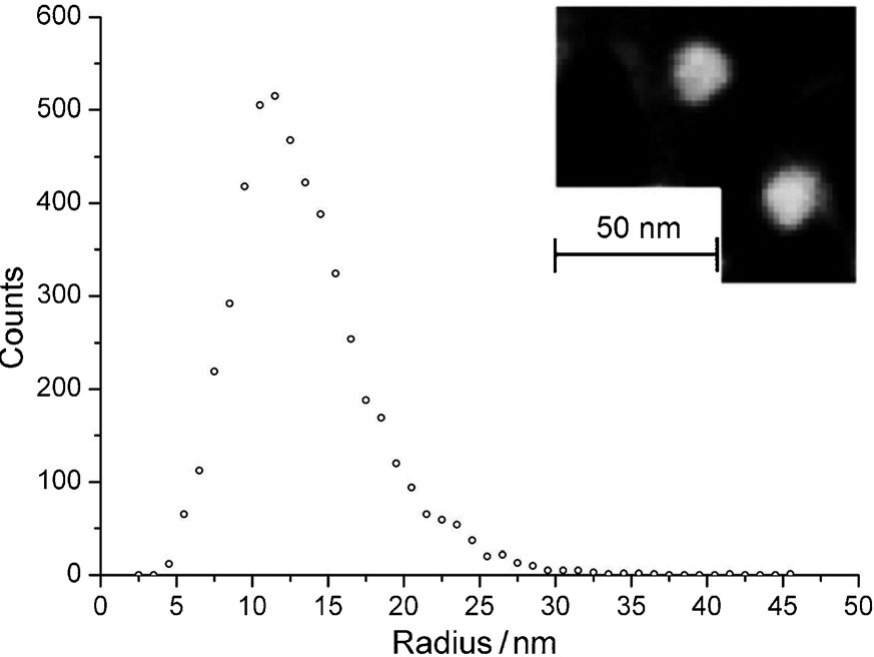
Size distribution of the Ag NPs used in this work (in the stock suspension) as determined by nanoparticle tracking analysis (NanoSight) and SEM image of Ag NP monomers (inset).

Thus, the number of silver atoms per NP is proportional to 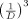 and, as for the impact data this number of atoms is directly available from the NTA measurements, as long as the shape of the considered particle is close to a sphere. However, for agglomerates of few monomers, the assumption of a spherical shape is not fully appropriate, as shown schematically in Figure 5. While monomers likely represent almost perfect spheres (see Figure 7 in the Experimental Section), dimers more precisely have to be described as dumbbells. Trimers and tetramers also form nonspherical agglomerates.

Consequently, the diffusion coefficients, which actually are the physically meaningful experimental output of NTA measurements, need to be corrected to yield detailed information about particle agglomeration states. Hoffmann et al.^13^ determined the required correction factor for agglomerates consisting of up to four monomers both theoretically and experimentally and found excellent agreement for both approaches. Accordingly, we derived the diffusion coefficients of agglomerates (*D*_*k*M_) from the value of the monomer (*D*_M_) by applying the correction factors (*α_k_*)


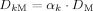
(12)

For the agglomerates shown in Figure 5, these factors were reported by Hoffmann et al.^13^ for dimers (*α*_2_=0.73), trimers (*α*_3_=0.64) and tetramers (*α*_4_=0.57), and we extrapolated these correction factors (see Figure 5 b) to correct for pentamers (*α*_5_=0.52) and hexamers (*α*_6_=0.50). In the following, these shape-corrected diffusion coefficients are used to fit the NanoSight data in light of agglomeration studies.

For better comparability with the impact data, we plotted the NTA data as counts against 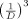, since this is directly proportional to the number of atoms plotted at the x-scale in Figure 3, which is not true for the diffusion coefficient (*D*) itself. Please note that this means we consider the actual measured data obtained by the NTA, not introducing any restrictions or assumptions with respect to the shape or nature of the NPs.

**Figure 6:**
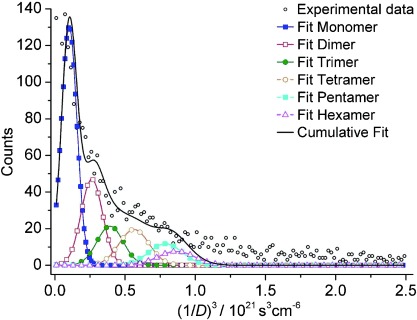
Experimentally detected distribution of the NP size in KCl/citrate electrolyte, as detected by NTA (○). The data fit (—) is derived by overlying the | individual Gaussians for NP monomers (▪), dimers (□), trimers (•), tetramers (○), pentamers (▪) and hexamers (▵).

Likewise to fitting the impact data (see above), the resulting counts against 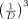 values is a convolution of *k* Gaussians with means at (1/*D*_*k*M_)^3^, which can be deconvoluted using the shape-corrected diffusion coefficients (see Figure 6). As for the fitting of the impact data, free fitting parameters only were the diffusion coefficient of the monomer (*D*_M_), the FWHM of the corresponding Gaussian (the standard deviation *σ*_M_) and the peak heights, which describe the relative concentration of the monomer and the different agglomerates in the electrolyte. The Gaussians of the individual agglomerates are given by their


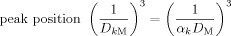
(13)


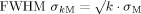
(14)

since again they follow a normal distribution (like the number of atoms, to which 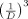 is directly proportional).

The fitted value for 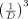 is 0.102×10^2^ s^3^ cm^−6^, that is, *D*_M_=2.14×10^−7^ cm^2^ s^−1^, which according to [Eq. (10)] corresponds to a monomer radius of 10 nm. Considering the precision of NanoSight data, which according to the producer is about 10 % of the NP size, this value is in excellent agreement with the value determined by the impact experiments (*r*_M_=11 nm), proving that both techniques are suitable for characterisation of NP sizes and agglomeration states independently of each other. To gain precise information about the latter, the new approach suggested herein, which considers the stochastics of experimental data and the nonspherical shape of formed agglomerates, is necessary.

### Agglomeration state of NPs in KCl/citrate

Besides analysing the size of the monomer and the various agglomerates present in the analysed electrolyte, it is also possible to quantify the relative concentration of monomers and agglomerates, taking the corresponding fitted peak intensities into account. For the impact experiments, these intensities directly display the number of detected impacts of NP of this specific kind. To derive the actual agglomeration state, that is, the ratio of the agglomerates, from this intensity ratio, the data has to be weighted by the respective diffusion coefficient (*D*_*k*M_). This weighting is necessary since impacts occur due to Brownian motion of the NPs, and the smaller the NPs are, the faster they move and the more likely they are to impact at the electrode within the duration of an experiment. Consequently, smaller NPs will be counted more frequently than larger NPs, with the corresponding diffusion coefficient being the weighting factor. To correct for this, the experimentally detected intensities were divided by the shape-corrected diffusion coefficients (*D*_*k*M_, see [Eq. (11)]). The resulting distribution of aggregation states in a KCl/citrate solution are summarised in Table 1. Thus, the impact data shows that only about 40 % of the NP in the solution are monomers, whereas one quarter of them are dimers and the remaining part are agglomerates of at least three monomers.

**Table 1:** Agglomeration state and relative concentrations of the NP monomers and agglomerates, as detected by impact and NTA experiments, with and without weighting by the corresponding diffusion coefficients (*D*_*k*M_).

No. monomers (*k*) in NP	Impact (fit)	Impact (*D*_*k*M_)	NanoSight (fit)	NanoSight (*D*_*k*M_)
1	49 %	38 %	55 %	43 %
2	23 %	24 %	20 %	22 %
3	10 %	12 %	9 %	11 %
4	7 %	10 %	8 %	11 %
5	7 %	10 %	5 %	8 %
6	4 %	6 %	3 %	5 %

Deriving the distribution of the various kinds of NPs from the NTA measurements also requires an additional weighting of the fitted intensity values. This weighting is automatically done by the implemented software for standard analysis. However, this does not take the nonspherical shape of agglomerates into account; thus, an alternative weighting needs to be applied. Using the track length, required for detected NPs to be considered in the statistics, was not appropriate for the present size distribution, since the automatically adjusted required minimum track length was 5 for all agglomerates. Taking into consideration that as for the impact experiments, diffusive motion of the NPs is crucial for them to be detected,^2^, ^13^ it seems reasonable to apply a similar weighting of the intensities fitted to the number of NP counted during the measurement, that is, dividing them by the shape-corrected value of the corresponding diffusion coefficient (*D*_*k*M_). Doing so yields a distribution of the agglomeration state that is very similar to the one determined by the impact experiments. However, it has to be noted, that while this weighting was physically straight-forward for the impact experiments, it might introduce an unknown error to the NanoSight data.

## Conclusion

In this work, we demonstrate that the simple experimental method of anodic particle coloumetry (APC) cannot only be used to size nanoparticles, but additionally allows the determination of their agglomeration state in a solution of interest. For this purpose, the impact data was analysed considering the link between the underlying normal distribution of the monomer and the formed agglomerates. Thus, the experimental data was fitted introducing as little free parameters as possible, hence preventing the loss of physical meaning during the fit. The obtained results were validated by a commercially available nanoparticle tracking system, and both techniques were in excellent agreement, proving the appropriateness of the suggested new approach of analysing APC data.

To the authors’ knowledge, this is the first time nanoparticle impact experiments have been analysed considering the link between the underlying normal distribution of the monomer and the formed agglomerates to gain precise information about the aggregation state of nanoparticles (NPs) in a specific electrolyte. Since, in contrast to nanoparticle tracking analysis (NTA) systems, the electrochemical method is not limited in terms of highly corrosive or optically nontransparent fluids, this new approach to nanoparticle agglomeration studies can be of superior interest for investigations of NPs. Further considering the flexibility of the electrochemical setup, which only requires three electrodes of micrometer scales, this opens new paths towards in situ aggregation studies in environmental systems.

## Experimental Section

**Chemicals**: Nanoparticle impact experiments were performed to analyse the size and the agglomeration state of silver nanoparticles (Ag NP) in an aqueous solution of KCl (0.09 m) and NaC_6_H_7_O_7_ (sodium citrate; 0.01 m). The latter was used for all electrochemical and nanoparticle tracking experiments, unless otherwise stated. All solutions were prepared from ultrapure H_2_O (Millipore, resistivity ∼18.2 MΩ cm at 25 °C) and chemicals were purchased from Sigma–Aldrich in analytical grade.

**Silver nanoparticles**: Citrate-capped Ag NPs were synthesised and washed according to^15^ and redispersed in ultrapure H_2_O containing NaC_6_H_7_O_7_ (1×10^−6^ m) to prevent agglomeration. The NP shape and size was characterized by high-resolution scanning electron microscopy (SEM; LEO Gemini 1530, Zeiss). Due to the strong tendency of NPs to agglomerate when drop cast onto a SEM holder, the Ag NP stock solution was diluted with H_2_O and drop cast onto a TEM-grid-modified SEM holder to enable imaging of non-agglomerated nanoparticles. The SEM image given in the inset of Figure 7 shows Ag NP monomers of spherical shape and a radius of about 10 nm. Statistics regarding the size were not possible, since agglomeration could not be prevented completely during preparation of the SEM sample and only few monomers were found. Hence, the size of NPs in the stock suspension was additionally determined by nanoparticle tracking analysis (Nanosight LM 10, NanoSight Ltd.) to a radius of *r*=11 nm (see Figure 7 black circles).

**Electrochemical analyses**: Electrochemical impact experiments were performed in a three-electrode setup at 20 °C employing a μAutolab II potentiostat (Metrohm-Autolab BV, Utrecht, Netherlands). A carbon fibre microelectrode (diameter=12 μm) was used as the working electrode (WE) and a graphite rod electrode (diameter=3 mm) served as the counter electrode. All potentials were applied with respect to a saturated calomel reference electrode (SCE, potential *E*=0.244 V versus standard hydrogen electrode) and are referenced to SCE throughout this article.

Voltammetric scans were performed to identify a suitable potential for anodic particle coloumetry (APC), that is to find a potential that suggests oxidation of Ag NPs at the WE. Therefore, a macro glassy carbon (GC) electrode was modified with Ag NPs by drop casting 3 μL of the Ag NP stock suspension onto it and drying it under N_2_. Linear sweep voltammograms of Ag NP stripping were recorded from −0.1 V to 0.6 V with a scan rate of 0.02 V s^−1^.

For the impact experiments, the Ag NP stock suspension (1 mL) was added to the electrolyte (17 mL). Chronoamperograms were recorded at 0.3 V to assure complete oxidation of the impacting Ag NPs and thus to enable their sizing by anodic particle coloumetry (APC).^5^ To improve the impact statistics, chronoamperograms were run over 5 s and repeated for 115 times, yielding a total number of 1333 impacts. The in-house developed software SignalCounter was employed for peak identification, baseline correction and determination of peak areas. Additional data treatment and curve fitting were done using Origin Pro 8.5.1 (Origin Lab Corporation).

**Nanoparticle tracking analysis**: Nanoparticle tracking analysis (NTA; NanoSight LM 10, NanoSight Ltd. Amesbury, UK) was used to determine the agglomeration state of the Ag NPs in the KCl/citrate solution. Therefore, the Ag NP stock suspension (1 mL) was added to KCl/citrate solution (17 mL), and the suspension (3 μL) was injected into the NanoSight measurement chamber using a syringe. Particles were illuminated by a red laser (wavelength=638 nm) and were tracked over a time period of 30 s. Data processing was performed using the integrated software package (NTA 2.2 build 0366), including the temperature of the electrolyte measured during the analysis (20 °C). Two consecutive scans were performed, then the measurement cell was cleaned and fresh NP–electrolyte suspension was injected for subsequent measurements. In total, 6 scans were recorded and 3050 NP tracks were analysed. Additional data treatment and curve fitting was done using Origin Pro 8.5.1 (Origin Lab Corporation).

## Footnotes

1 According to the IUPAC definition agglomeration and aggregation refer to a reversible and irreversible sticking of particles, respectively.^10^ The analysis techniques referred to in this work detect both simultaneously. Therefore, throughout the article only the expression agglomeration will be used to refer to both types of particle adhesion for mutual convenience.
